# Medical Opinion and Movement

**Published:** 1909-12-25

**Authors:** 


### Medical Opinion and Movement.

In the delivery of a healthy infant it is the usual practice now to delay Division and Ligature of the cord till after pulsation has ceased, so as to allow of the passage of an extra amount of blood from the maternal organism to the infant. But in the case of a child born apparently dead the custom is rather to divide and ligature the cord at once, so as to be able better to proceed to means of revivifying the infant. Dr. J. Jerie, of Prague, suggests, however, that this is a mistake, and that the extra supply of oxygenated blood from the maternal organism may be an important and useful factor in the reanimation of the infant. He gives details of three cases of infants born in a state of serious asphyxia, in which he resorted to artificial respiration with the cord still intact. After a very few movements the pulsations of the cord, at first feeble, became much stronger, and the signs of asphyxia rapidly disappeared. The idea is certainly reasonable, and deserves to be borne in mind in such cases.

---

The administration of Adrenalin in normal saline solution by intravenous or subcutaneous injections is a well-recognised form of treatment in conditions of surgical collapse and shock, especially in conditions of splanchnic hypotension after abdominal operations. The use of adrenalin for this purpose was originally suggested by Heidenhain, and has since been advocated by Crib. In view of the good effects in these conditions Dr. M. John, of Mannheim, has tried adrenalin injections in cases of collapse of cardiac origin, and is well satisfied with the results. He has treated altogether 30 cases of severe collapse, in all of which other more usual means had been tried, such as hypodermic injections of caffeine, camphorated oil, intravenous injections of strophanthus, etc., and the adrenalin has always proved efficacious. He gives a warning, however, against its use in cases of cardiac insufficiency secondary to granular kidney, and reports a case in which death followed intravenous injection of a small dose of a 1 in 1,000 adrenalin solution, although the patient had reacted well to two previous similar doses. At the autopsy the kidneys were found to be of the small granular type. In other cases the injections may be repeated if necessary in four to six hours, or on the following day, according to the condition of the patient.

---

At a meeting of the *Société Médicale des Hôpitaux* Dr. Menetrier gave an interesting account of the effect of the Röntgen Rays upon diabetic patients when applied to the hepatic region. The effect is most marked in the more severe form of the disease, with general debility and loss of flesh. In these cases the application of the $x$-rays is followed by a considerable increase in the glycosuria within the next 24 hours, and sometimes for several subsequent days, and a fall in the number of red blood corpuscles. Thus in one case the amount of sugar excreted in 24 hours rose from 1,000 to 1,600 grammes, and the number of red corpuscles fell from 3,470,000 to 1,170,000, or a loss of 2,300,000 corpuscles per cubic millimetre. The subsequent effect is, however, just the inverse of the preceding, and after some days of increased sugar elimination the quantity steadily diminishes till it is below the amount originally excreted before the $x$-ray applications. Similarly the loss in blood corpuscles is rapidly repaired, and the number of red corpuscles in the blood after successive applications of the $x$-rays is soon higher than the original count. With diabetics of the obese type with only slight glycosuria these different effects are much less marked. Experimenting with guinea pigs, it has been found that the application of $x$-rays in large doses causes a complete disappearance of glycogen in the liver, accompanied by a marked atrophy of that organ.

---

An ingenious research into the best method for Opening the Kidney in the operation of Nephrotomy has been carried out by Drs. Cullen and Derge, whose preliminary report is published in the *Johns Hopkins Hospital Bulletin*. The experimenters were impressed with the conviction, after studying Brödel's demonstration of the arborescent branchings of the renal arteries from pelvis to cortex, that to minimise hæmorrhage and damage to vascular structures generally in splitting the kidney it would be better to divide the organ from the pelvis outwards rather than the other way; by this means many of the fine vessels would be brushed aside instead of being cut or torn. They further concluded that a slightly blunt instrument should be better adapted to this end than a very sharp one. Accordingly they divided a dog's kidney from cortex to pelvis, and another the opposite way, with a knife. In the first case 16.5 c.c. of blood were lost in 20 seconds; in the second only 11 c.c. Then they tried silver wire by comparison with the knife, and these sections were made not only from pole to pole parallel with the sagittal plane of the kidney, but also in planes at right angles to its curved vertical axis. A silver wire was introduced and made to traverse the kidney somewhat in the manner of a cheese-cutter; firm counter-traction on the kidney is necessary to prevent tearing of the renal vessels. The results are described as excellent. The kidney can be thus opened almost without loss of blood at all, whereas when similar incisions were produced with a knife blood spurted freely, and the loss was two to nine times as great. No special effort is made to direct the wire, which is brought out in the plane of least resistance; this results in the hitting off of Brödel's avascular line. Mere approximation of the cut surfaces without suture suffices to check any slight oozing there may be; but the authors advise superficial suture to maintain approximation. The method is being tested in man, with results which so far have been highly satisfactory.

